# Motor Imagery as a Function of Disease Severity in Multiple Sclerosis: An fMRI Study

**DOI:** 10.3389/fnhum.2017.00628

**Published:** 2018-01-11

**Authors:** Andrea Tacchino, Catarina Saiote, Giampaolo Brichetto, Giulia Bommarito, Luca Roccatagliata, Christian Cordano, Mario A. Battaglia, Gian L. Mancardi, Matilde Inglese

**Affiliations:** ^1^Scientific Research Area, Italian MS Foundation (FISM), Genoa, Italy; ^2^Department of Neurology, Icahn School of Medicine at Mount Sinai, New York, NY, United States; ^3^Department of Psychiatry, Icahn School of Medicine at Mount Sinai, New York, NY, United States; ^4^Department of Neuroscience, Rehabilitation, Ophthalmology, Genetics and Maternal and Child Health (DINOGMI), University of Genoa, Genoa, Italy; ^5^Department of Health Sciences (DISSAL), IRCCS San Martino University Hospital and IST, Genoa, Italy; ^6^Neuroradiology Department, IRCCS San Martino University Hospital and IST, Genoa, Italy; ^7^Department of Life Science, University of Siena, Siena, Italy; ^8^Department of Radiology, Icahn School of Medicine at Mount Sinai, New York, NY, United States; ^9^Department of Neuroscience, Icahn School of Medicine at Mount Sinai, New York, NY, United States

**Keywords:** motor imagery, fMRI, isochrony, multiple sclerosis, disease severity, disease marker

## Abstract

Motor imagery (MI) is defined as mental execution without any actual movement. While healthy adults usually show temporal equivalence, i.e., isochrony, between the mental simulation of an action and its actual performance, neurological disorders are associated with anisochrony. Unlike in patients with stroke and Parkinson disease, only a few studies have investigated differences of MI ability in multiple sclerosis (MS). However, the relationship among disease severity, anisochrony and brain activation patterns during MI has not been investigated yet. Here, we propose to investigate MI in MS patients using fMRI during a behavioral task executed with dominant/non-dominant hand and to evaluate whether anisochrony is associated with disease severity. Thirty-seven right-handed MS patients, 17 with clinically isolated syndrome (CIS) suggestive of MS and 20 with relapsing-remitting MS (RR-MS) and 20 right-handed healthy controls (HC) underwent fMRI during a motor task consisting in the actual or imaged movement of squeezing a foam ball with the dominant and non-dominant hand. The same tasks were performed outside the MRI room to record the number of actual and imagined ball squeezes, and calculate an Index of performance (IP) based on the ratio between actual and imagined movements. IP showed that a progressive loss of ability in simulating actions (i.e., anisochrony) more pronounced for non-dominant hand, was found as function of the disease course. Moreover, anisochrony was associated with activation of occipito-parieto-frontal areas that were more extensive at the early stages of the disease, probably in order to counteract the changes due to MS. However, the neural engagement of compensatory brain areas becomes more difficult with more challenging tasks, i.e., dominant vs. non-dominant hand, with a consequent deficit in behavioral performance. These results show a strict association between MI performance and disease severity, suggesting that, at early stages of the disease, anisochrony in MI could be considered as surrogate behavioral marker of MS severity.

## Introduction

Motor imagery (MI) is defined as a dynamic state during which the representation of a specific motor action is internally reactivated within working memory without any actual motor output (Jeannerod, 1995). This phenomenological experience is governed by the principles of central motor control and consists in a first-person perspective of performing a movement.

The mentally and actually executed movements share common characteristics (Grèzes and Decety, 2001; Jeannerod, 2001; de Lange et al., 2005, 2008). Studies of fMRI have demonstrated that both motor execution and MI activate brain regions involved in motor planning and execution, including the supplementary motor area (SMA), premotor area (PMA), primary sensorimotor area (M1/S1), posterior parietal lobe (PPL), striatum, cerebellum and thalamus, although at different volume and intensity of activation (Lotze et al., 1999; Gerardin et al., 2000; Lacourse et al., 2005; Munzert et al., 2009; Zhang et al., 2012; Saiote et al., 2016).

From a behavioral standpoint, the required time to perform a specific movement is tightly correlated with the required time to mentally simulate the same movement. It has been shown that healthy adults show temporal equivalence, i.e., isochrony, between the mental simulation of an action and its actual performance (Gentili et al., 2004; Guillot and Collet, 2005; Munzert et al., 2015). Conversely, the temporal uncoupling between actual and mental movements, i.e., anisochrony, may be the consequence of either abnormal developmental processes (Choudhury et al., 2007; Caeyenberghs et al., 2009) or structural and functional network changes in neural networks occurring with aging (Skoura et al., 2008; Personnier et al., 2010) and with neurological disorders (Sirigu et al., 1996; Daprati et al., 2010; Abbruzzese et al., 2015).

However, there is increasing evidence that the effect of mental practice is partially preserved in neurological diseases such as stroke and Parkinson disease (Sharma et al., 2006; Tamir et al., 2007; Zimmermann-Schlatter et al., 2008) supporting the use of MI in rehabilitative protocols aimed at re-activating sensorimotor networks and promoting motor recovery (Jackson et al., 2001; Johnson et al., 2002; Sharma et al., 2006; de Vries and Mulder, 2007).

Unlike in patients with stroke and Parkinson disease, only few studies have investigated differences of MI ability in multiple sclerosis (MS) and showed that patients had a significantly lower accuracy score than controls in correctly executing a hand rotation task (Heremans et al., 2012; Tabrizi et al., 2013). A recent work from our group confirmed the presence of anisochrony between actual and mental movement in MS patients during a motor task with the upper limbs. We showed that actual movement duration was significantly longer than mental movement duration for the non-dominant arm and suggested that these differences could be related to increased cognitive effort required for performing movements with the non-dominant limb (Tacchino et al., 2013). None of these studies, however, investigated the neural correlates of MI impairment in MS. Both focal and diffuse MS damage of white and gray matter could contribute to the development of motor and cognitive deficits, thus affecting alteration of temporal processing of imagined actions. In addition, although it is known that motor-cognitive impairments accumulate with disability progression (Johansson et al., 2007) affecting movement planning and online control it is still not clear whether anisochrony between MI and motor execution in MS patients is associated with the disease severity. The investigation of the neural correlates of MI as function of disease severity could improve our understanding of changes in behavioral performance (i.e., anisochrony) and could contribute to the development of more effective motor-cognitive strategies to be employed in rehabilitative interventions.

Therefore, the aims of our study were: (a) to investigate the neural correlates of MI in patients with MS using fMRI during a task executed with the right and left hands; (b) to explore the association among disease severity, anisochrony and brain activation patterns during MI. In particular, we focused our attention on the first stages of the disease, by comparing two populations of MS patients, clinically isolated syndrome (CIS) and relapsing-remitting (RR-MS), to healthy controls (HC) matched for age and educational level.

CIS is the initial presentation in 80% of MS cases. CIS encompasses an acute clinical attack affecting one or more central nervous system sites and converts to RR-MS with a variable rate, from 20% to 80% depending on the presence and the number of clinically silent white matter lesions on MRI. These early stages of MS are characterized by demyelination processes, thought to have a common inflammatory pathological substrate (Doshi and Chataway, 2016). Although, there is usually good recovery from an attack, a decreased whole-brain network efficiency was observed for both CIS and RR-MS patients. Disrupted network organization already emerges in CIS, even if with a lesser degree relative to MS, suggesting that differences could be present between populations in mentally predicting motor actions (Liu et al., 2017).

## Materials and Methods

### Participants

Patients with CIS suggestive of MS and clinically definite MS (Polman et al., 2011) with RR-MS course (Lublin et al., 2014) were prospectively recruited from the outpatients clinic of the DINOGMI (Department of Neuroscience, Rehabilitation, Ophthalmology, Genetics, Maternal and Child Health, University of Genoa, Italy). To be enrolled, patients had to be in a stable phase of the disease without relapses in the 3 months prior to study entry and present with mild disability of the upper limbs. Sex- and age-matched healthy volunteers served as controls. All participants had to be right-handed as determined by the Edinburgh Handedness Inventory Scale (EDSS; score > 90%). For all participants, the exclusion criteria were: (a) history of major systemic, neurologic or psychiatric disorder; (b) contraindications to MRI.

This study was carried out in accordance with the recommendations of COMITATO ETICO AZIENDALE A.O. UNIVERSITARIA “SAN MARTINO” GENOVA with written informed consent from all subjects. All subjects gave written informed consent in accordance with the Declaration of Helsinki. The protocol was approved by the COMITATO ETICO AZIENDALE A.O. UNIVERSITARIA “SAN MARTINO” GENOVA.

### Experimental Design

On the same day of MRI acquisition patients underwent neurological evaluation with EDSS rating, and all subjects were administered the Modified Fatigue Impact Scale (MFIS; Flachenecker et al., 2002) for assessment of fatigue, the Symbol Digit Modalities Test (SDMT; Andreasen et al., 2010) for assessment of cognitive dysfunction, the Nine-Hole Peg Test (9HPT; Fischer et al., 1999) for assessment of upper limb motor-sensory status and the Kinesthetic and Visual Imagery Questionnaire (KVIQ; Malouin et al., 2007) in the first person perspective (Stevens, 2005) for evaluating imagery ability.

### Actual Execution and MI Tasks

Before undergoing fMRI, all subjects were asked to actually and mentally perform a simple motor task in order to assess eventual differences in isochrony among groups. This step took place in a quiet and sound-attenuated room and the participants were comfortably seated on an adjustable chair. The motor task consisted in a squeezing ball movement with the dominant and non-dominant hand (Mizuguchi et al., 2013). During the actual squeezing ball movement (AM), subjects were invited to hold a foam ball of 7 cm diameter with the hand and requested to squeeze the ball repetitively at the self-paced speed. During the mental squeezing ball movement (MM), subjects had to keep the ball in their hand as in the previous task but they were requested to imagine squeezing it repetitively at a self-paced speed in the first person perspective. In both conditions, they were instructed to maintain the same pace during the experiment. Before recording, all the subjects were trained to actually and mentally squeeze the foam ball several times at their own pace (Mizuguchi et al., 2013).

Both conditions were performed with the right and left hand for a total of four tasks (AM_R, MM_R; AM_L, MM_L); the hand order was randomly presented. Each task consisted of four consecutive runs of 60-s: 30 s of rest followed by 30 s of AM (for the actual execution tasks) or MM (for the MI tasks). The participants rested or executed AM or MM following an *ad hoc* computer presentation of temporized red and green slides. Red signaled rest whereas green indicated to perform AM or MM.

The motor tasks were performed outside the magnet room due to the difficulty in recording the number of mental or actual ball squeezes during fMRI. The executed ball squeezes were counted by the operator, while imagined ball squeezes were mentally counted and reported by the subject at the end of each task. After this step, fMRI experiment started and the same motor tasks were repeated.

For each of the four tasks the average of the number of squeezes of the four consecutive runs was calculated. Then, the ratio (AM/MM) was calculated for both hands separately and used to define the Index of performance (IP) as (1-AM/MM), according to Saiote et al. (2016). Thus, an IP of 0 indicates very good precision (i.e., isochrony), while an IP < 0 or IP > 0 denotes anisochrony due to a number of mental squeezes smaller or greater than the corresponding actual (mental task slower or faster than the actual task), respectively.

### MRI Acquisition

All subjects underwent MRI at 1.5T (SignaHDxt scanner, GE MEDICAL Systems), using an 8-channel phased array head coil. The MRI protocol included the following sequences: (a) axial PD-T2-weighted (TR/TE1/TE2 = 2340/102/38.25 ms; FA = 90°; voxel size = 0.94 × 0.94 × 4 mm^3^); (b) sagittal 3DT1-weighted FSPGR (TR/TE/TI = 11.56/5.048/500 ms; FA = 8°, voxel size = 1 × 1 × 1 mm^3^); (c) gradient-echo EPI with 83 volumes for each of the four task-fMRI runs (TR/TE = 3000/60 ms; FA = 90°, slice spacing = 1 mm, voxel size = 3.75 × 3.75 × 4 mm^3^).

### Structural MRI Analysis

White matter lesion volume was measured on T2-weighted images using Jim (version 3; Xinapse Systems, Northamptonshire, UK) as previously described (Inglese et al., 2010). Masks of the white matter lesions identified on the T2-weighted images were used to evaluate the presence of focal lesions within the cortico-spinal tract (CST). For each patient, T2-weighted images were linearly co-registered to the native T1-weighted space and, subsequently, the transformation matrix was applied to the lesion mask, using FLIRT (Jenkinson et al., 2002). Lesion and CST masks were visualized in T1 space to obtain the lesion number within each tract.

### fMRI Analysis

Analysis of fMRI was performed as described in Saiote et al. (2016). Briefly, preprocessing included slice-timing correction for regular ascending acquisition (with Fourier-space time series phase-shifting) and despiking (detection and reduction of extreme time series outliers by fitting a smooth curve insensitive to extreme outliers to the data) were performed in AFNI^1^. Then, removal of the first three volumes, motion correction using MCFLIRT (Jenkinson et al., 2002), brain extraction using BET (Smith, 2002), spatial smoothing (Gaussian kernel, FWHM = 6 mm), grand-mean intensity normalization of all volumes by a single multiplicative factor, and high-pass temporal filtering (Gaussian-weighted least-squares straight line fitting, sigma = 30 s) were performed using FEAT (Woolrich et al., 2001, 2009), part of FSL (Smith et al., 2004; Jenkinson et al., 2012).

For the first-level individual general linear model analysis, the On-Off blocks of the task were convolved with the hemodynamic response function and defined as the explanatory variable of interest. Mean signal from white matter and CSF was calculated by segmenting T1-weighted images with FAST, then registering the resulting white matter and CSF masks to functional space and, finally, averaging the raw time series within each mask. These were added as confound explanatory variables as well as the six motion parameters calculated during motion correction. One contrast was defined to obtain individual maps of activation related to each of the four tasks: AM_R, AM_L, MM_R and MM_L. For higher-level analysis, mixed-effects models as implemented in FSL FLAME were used to model one-sample *t*-tests to determine the group mean for each task. Two-sample paired *t*-tests were used to obtain group comparisons of AM and MM, separately for each hand, with age and gender added as covariates. Additionally, whole-brain voxelwise correlations between MM activation and the IP were performed for the whole group of patients, and separately for RR-MS and CIS patients keeping age and gender as covariates. Statistical maps were masked for gray matter voxels using a gray matter mask (derived from the MNI template GM probability map thresholded at 0.26 probability). Results were corrected for multiple comparisons with Gaussian random field theory (cluster threshold *Z* > 2.3; *p* < 0.05).

### Statistical Analysis

Statistical analysis was computed using STATISTICA 7.1 (StatSoft, Tulsa, OK, USA). To evaluate if data were normally distributed the Shapiro-Wilk test was performed for IP and all demographic (age and educational level) and behavioral (EDSS, SDMT, 9HPT, MFIS and KVIQ) variables and for each group separately. ANOVA or nonparametric Mann-Whitney test were used for between-group comparisons for each variable. *Post hoc* differences were assessed by means of Newman–Keuls tests, and the level of significance was set at *P* < 0.05. A linear regression model was applied to evaluate the relationship of KVIQ, right and left IP with the other behavioral variables (right and left 9HPT, MFIS and SDMT). Linear correlation was used to better characterize the groups differences between behavioral parameters and MRI metrics.

## Results

### Participants

Thirty-seven MS patients, 20 with RR-MS (9 males and 11 females; mean age 39.10 ± 9.45 years; educational level 13.90 ± 3.58 years; mean disease duration 27.80 ± 15.45 months) and 17 with CIS (7 males and 10 females; mean age 35.53 ± 8.16 years; educational level 14.59 ± 4.17 years; mean time from the attack 14.12 ± 8.20 years), and 20 HC (8 males and 12 females; mean age 33.95 ± 8.08 years; educational level 16.30 ± 2.58 years) were prospectively enrolled. Age and educational levels were normally distributed and there was no statistically significant difference between the three groups of subjects (ANOVA: *P* = 0.17 and *P* = 0.09 respectively). The EDSS score was significantly higher in RR-MS patients (median: 1.5, range: 1.0–3.5) than in CIS patients (median: 1.0, range: 0.0–2.0; Mann-Whitney test: *P* < 0.01). The ANOVA test, corrected for age, gender and educational level for SDMT, MFIS, 9HPT and KVIQ, did not show any statistically significant difference among groups. Groups showed similar values of SDMT (mean 54.20 ± 15.02 for RR-MS; 58.94 ± 10.74 for CIS; 58.60 ± 10.67 for HC) and MFIS (mean 24.00 ± 18.39 for RR-MS; 15.18 ± 10.77 for CIS; 12.35 ± 8.94 for HC). The 9HPT score was significantly longer (*P* < 0.05) in the RR-MS (mean 21.36 ± 4.50 s) than in the CIS (mean 18.93 ± 2.99 s) and HC (mean 18.84 ± 2.11 s; *F*_(2,52)_ = 3.30, *P* < 0.05) group. All subjects rated their imagery vividness as good (mean KVIQ score: 77.50 ± 15.54 for RR-MS; 77.59 ± 17.59 for CIS; 77.40 ± 14.31 for HC). No significant relationships between KVIQ and behavioral variables were found, for each single group, for all the patients if considered as a single group (RR-MS and CIS together) and for all participants together (RR-MS, CIS and HC) as demonstrated by the linear regression model.

As expected, given the different disease course and duration of the RR-MS and CIS groups, T2 hyperintense lesion volume (T2LV) was significantly higher in RR-MS (mean: 8.0, SD: 8.3) compared to CIS patients (mean: 1.8, SD: 1.7; *P* = 0.001). Moreover, the number of lesions in the CST was significantly higher in RR-MS (mean: 0.68, SD: 0.94) compared to CIS patients (mean: 0.12, SD: 0.33; *P* = 0.019). A positive correlation between the number of lesions in the left CST and right 9HPT (*P* = 0.002) and between right CST and left 9HPT (*P* = 0.011) was found for RR-MS, whereas no correlations were found for CIS group.

### Index of Performance

The IP values revealed that the number of imaged ball squeezes was smaller than the number of executed squeezes in the three groups of subjects (Supplementary Table S1). IP increased with the worsening of the disease course revealing a progressive anisochrony in the ability to simulate actions (RR-MS: right, 0.51 ± 0.48, left, 0.61 ± 0.52; CIS: right, 0.31 ± 0.22, left, 0.37 ± 0.29; HC: right, 0.20 ± 0.13, left, 0.20 ± 0.17). IP for the dominant and non-dominant hand was normally distributed in all the groups. ANOVA analysis, performed with group as a between-subject factor and hand as a within-subject factor, revealed a main effect of group (*F*_(2,54)_ = 5.86, *P* < 0.05) and hand (*F*_(1,54)_ = 5.01, *P* < 0.05). *Post hoc* analysis showed significant differences between RR-MS group (mean 0.56 ± 0.45) with respect to CIS (mean 0.34 ± 0.26) and HC (mean 0.20 ± 0.15; *P* < 0.05) independently of the hand and between right and the left hand (mean 0.34 ± 0.26 and 0.39 ± 0.30 respectively; *P* < 0.05) independently of the group. Although no significant interaction between group and hand was found, as reported in previous results (Tacchino et al., 2013), an increasing discrepancy in anisochrony between hands was found as function of the disease course with worsening of performance more pronounced on the non-dominant hand.

No significant relationships between right and left IP and behavioral variables and T2LV were found, for each patient group, or for all the patients (RR-MS and CIS together) or for all participants together (RR-MS, CIS and HC) as demonstrated by the linear regression model.

### fMRI Activation during AM and MM

**Figure 1:**
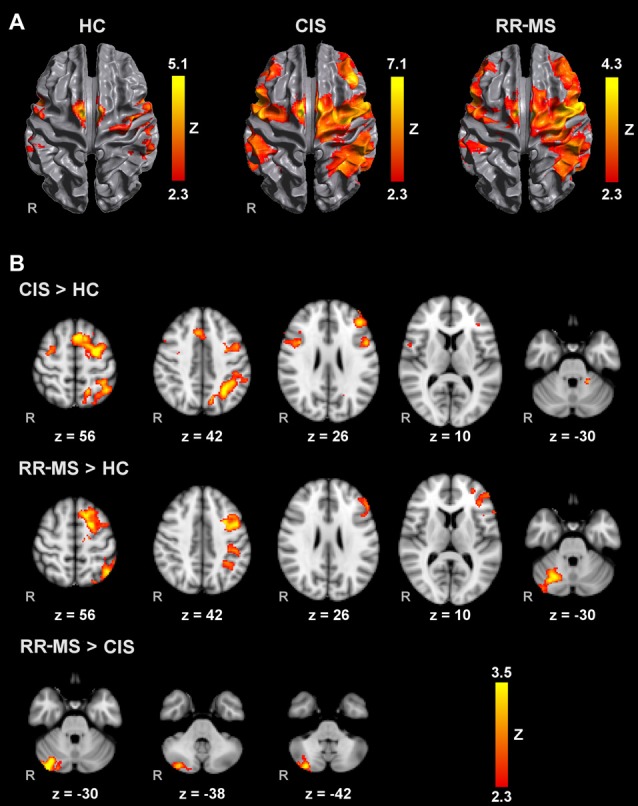
Brain activation during mental squeezing ball movement (MM) with the right hand **(A)** in healthy controls (HC), clinically isolated syndrome (CIS) and relapsing-remitting multiple sclerosis (RRMS) groups and **(B)** comparisons between groups. Results are cluster corrected for multiple comparisons (*Z* > 2.3; *P* < 0.05) and are shown overlaid on the MNI template.

**Figure 2:**
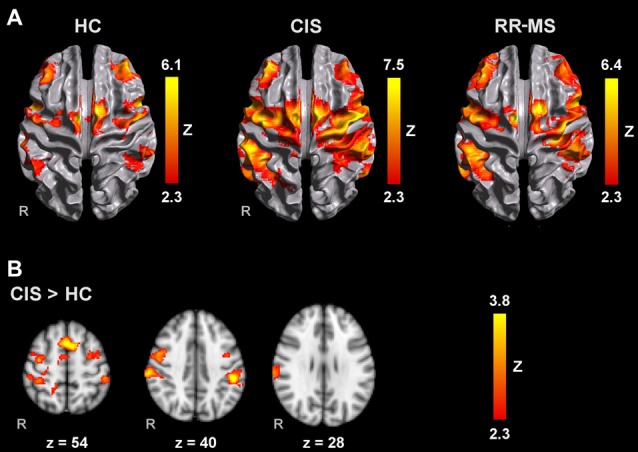
Brain activation during MM with the left hand **(A)** in HC, CIS and RRMS groups and **(B)** comparisons between groups. Results are cluster corrected for multiple comparisons (*Z* > 2.3; *P* < 0.05) and are shown overlaid on the MNI template.

All groups activated an extensive motor network during AM with the right and left hands, including the contralateral M1, S1 and superior parietal lobule (SPL), SMA, bilateral lateral PM, contralateral thalamus, bilateral putamen and cerebellum. Activated clusters were more extensive in RR-MS patients than CIS patients and HC, and in CIS patients compared to HC, in agreement with the literature (Pantano et al., 2005; Rocca et al., 2005; Lenzi et al., 2007). During MM, there was a recruitment of the same motor network although with less extensive M1 and S1 activation and additional activation of prefrontal and parietal regions (Figures 1A, 2A).

### Group Comparisons of Brain Activation during MM

Direct pairwise group comparison of MI brain activations during MM_R (Figure 1B), showed that compared to HC, CIS patients showed higher activation bilaterally in the SPL, anterior intra-parietal sulcus (IPS), left dorsal premotor cortex (PMd), left superior frontal gyrus (SFG) and middle frontal gyrus (MFG), left inferior and superior lateral occipital cortex (LOC) and pre-SMA.

Compared to HC, RR-MS patients presented higher activation in the left SPL, left anterior IPS, left PMd, left SFG, left inferior LOC and right cerebellum. Compared to CIS patients, RR-MS patients showed stronger recruitment of the right cerebellum. During MM_L, comparison between CIS and HC showed that CIS patients had a significantly higher recruitment of the right SPL, right S1, right M1 and bilateral PMd, and HC had higher recruitment of the left SFG and MFG, and bilateral superior LOC (Figure 2B). No other significant group differences were found.

### Comparison of MM and AM Differences between Groups

CIS patients showed significantly higher MM_R > AM_R activations than HC in several regions within the MI network, in bilateral parietal, premotor and prefrontal areas, pre-SMA, left insula, left putamen and right cerebellum. Similar results were found for MM_L > AM_L, with less extensive differences in the left hemisphere. CIS patients also showed significantly higher MM_L > AM_L than RR-MS group in the right S1 and IPS, pre-SMA and anterior cingulate cortex. In the RR-MS group, increased MM_R > AM_R differences in comparison with HC were found mainly in the left hemisphere, in the S1, premotor and frontal region, left putamen, and bilaterally in ventrolateral prefrontal cortex and cerebellum. No significant differences between RR-MS and HC were observed for MM_L > AM_L.

When taking into account disease severity using EDSS as a covariate, regions with increased activation during MM_R compared to AM_R were higher in CIS patients compared to both RR patients and HC.

### Voxelwise Correlations with the IP

**Figure 3:**
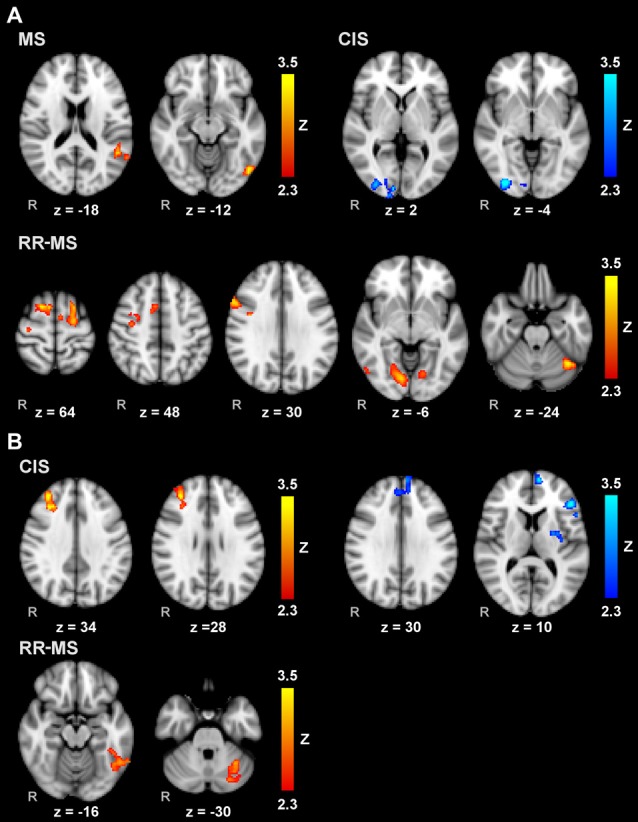
Voxelwise correlations of brain activity during MM with the right **(A)** and left **(B)** hands and the corresponding index of performance (IP) scores for the whole group of MS patients, and separately for CIS and RR-MS patients. Results are cluster corrected for multiple comparisons (*Z* > 2.3; *P* < 0.05) and are shown in red-yellow for positive correlations, indicating worse performance associated with more activation, and light-dark blue for negative correlations, indicating where better performance is associated with more activation, overlaid on the MNI template.

Voxelwise correlation analysis between IP and brain activity during MM_R (Figure 3A) revealed: (a) significant clusters correlating positively in the left inferior parietal lobule (IPL; angular gyrus, middle temporal gyrus and planum temporale) and left visual area V5 in the whole group of MS patients; (b) significant clusters positively correlating in the cerebellum and V3 bilaterally, right V5, right inferior frontal gyrus (IFG) and precentral gyrus, bilateral PMd, right MFG, bilateral SFG, left SMA and right paracingulate gyrus in RR-MS patients; and (c) negative correlations in CIS patients in the right visual areas V1 and V3.

Voxelwise correlation analysis between IP and brain activity during MM_L (Figure 3B) revealed: (a) significant clusters correlating positively in the right dorsolateral prefrontal cortex in CIS patients; (b) clusters correlating negatively in the left medial prefrontal cortex, left IFG, and left putamen and pallidum in the CIS patients; and (c) clusters correlating positively in the left inferior temporal gyrus and left cerebellum (VI and Crus I). There were no significant correlations with the whole MS patient group.

## Discussion

To the best of our knowledge, this is the first study that characterizes the neural correlates of MI in MS patients. Previous studies focused on the behavioral aspects of MI and did not explore the impact of disease severity on imagery ability (Heremans et al., 2012; Tabrizi et al., 2013; Tacchino et al., 2013).

Here, MI ability and corresponding neural correlates of RR-MS patients with mild disability, CIS patients and a group of healthy subjects were compared. Based on the IP, our results suggest that MI ability depends on disease severity as shown by the significantly higher anisochrony in RR-MS patients than CIS patients and controls. Our task consisted of simple squeeze ball movements to be repeatedly imagined in blocks of long duration (30 s). This design require prolonged attention that may have led to a slowdown in the mental motor simulation and a consequent overestimation of movement duration during mental execution (Sabaté et al., 2004; Stinear et al., 2007; Lebon et al., 2012). This might have been further stressed in our task because we asked participants to count how many movements they were performing during MI, which might increase the mental effort, forcing two different mental tasks taking place simultaneously. However, the strict temporal coupling between the two tasks (i.e., an increment in counting concomitant to each imagined movement) and the minimal memory and calculus resources demand (i.e., adding 1) let suppose that these effects should equally influence all the participants. Consequently, the significantly higher anisochrony in patients could be mainly explained by the impact of MS pathology on the forward internal models used in mental prediction of motor actions (Miall and Wolpert, 1996; Wolpert and Flanagan, 2001).

Task-dependency may also explain why here we found imagined movements slower than those actually executed, differently from our previous study (Tacchino et al., 2013). There, RR-MS patients had to perform overt and covert pointing tasks as accurately and as fast as possible between targets of several sizes. The results showed that they imagined faster than when the task was actually performed, probably as consequence of two coexistent causes: the difficulty of the task led to slowness in actual execution and patients probably adopted a simulation strategy that did not fully integrate task constraints (i.e., target size and movement speed) during the mental process, allowing to speed up the mental representation (Guillot and Collet, 2005).

No cognitive and fatigue differences were found, but worse fine hand movements were observed in RR-MS patients with respects to the other groups. The upper limbs physical effects due to MS progression, albeit significant, were probably not consciously perceived, remaining subtle (Solaro et al., 2007), and consequently did not affect a process of self-evaluation such as that required while they rated own imagery vividness through KVIQ (Heremans et al., 2012; Tabrizi et al., 2013; Saiote et al., 2016).

As expected, during actually executed tasks all subjects recruited a network of cortical and subcortical brain regions deputed to motor and sensory control that became more extensive in patients likely as a compensatory mechanism to maintain a normal performance despite the presence of focal and diffuse brain injury (Guillot and Collet, 2005; Pantano et al., 2005; Rocca et al., 2005; Lenzi et al., 2007). A similar pattern of activation was elicited during MI tasks, though M1 and S1 activations were less extensive due to a less essential role of the sensory-motor network during motor simulation and due to the minimal activation of the bulk of the thalamo-cortical projections from sensory inputs (Sharma et al., 2008; Munzert et al., 2009; Di Rienzo et al., 2014). Furthermore, during MI with the dominant hand, CIS and RR-MS patients showed an additional recruitment of frontal, parietal and occipital areas, which may represent a compensatory strategy to achieve a better functional performance (Mizuguchi et al., 2016). Indeed, the activation of the left SFG, generally involved in tasks requiring attention and working memory (Owen, 2000; du Boisgueheneuc et al., 2006; Li et al., 2013) may indicate an increased need of attentional and memory resources to produce accurate mental images (Pokryszko-Dragan et al., 2016). Moreover, the contribute of left SFG to motor tasks (Nachev et al., 2008; Chouinard and Paus, 2010; Martino et al., 2011) together with the higher recruitment of pre-SMA in CIS and PMd and right cerebellum in RR-MS patients suggests a loss of automatism in action simulation with the consequent request of major engagement of brain areas involved in motor control during mental tasks. Indeed, the frontal regions (i.e., pre-SMA) recruited by CIS patients are involved in “higher” level functions (i.e., movement ideation, visuomotor associations, time perception and discrimination, action intention representation and internally guided actions; Nachev et al., 2007), whereas frontal areas (i.e., PMd) preferentially recruited by the RR-MS patients are deputed to movement preparation and selection (Kantak et al., 2012). In addition, the right cerebellum is known to play an important role in coordinating fine motor movements such as those used in this study (Stoodley and Schmahmann, 2009).

Besides frontal areas, during MI with the right hand, both groups of patients showed increased recruitment of parieto-occipital regions such as the SPL and the IPS. The former receives a great deal of sensory inputs, participates in tactile localization and spatial orientation and plays a crucial role in visuomotor coordination especially in producing purposeful and skilled movements. Similarly, the anterior IPS is associated to visuomotor processes such as visual attention and visuomotor coordination, object manipulation and cross-modal transfer of object information between the visual and sensorimotor systems (Grefkes and Fink, 2005). Their increased activation, together with that of LOC, involved in shape object recognition, representation and processing, highlight their critical role in imaging somatosensory and visual aspects, in coding spatial properties and in predicting the temporal feature of the movement to be imagined. Also, bilateral and not only contralateral activation of SPL and IPS in CIS group seems to stress the crucial role of sensory information for these patients in producing accurate MI. Moreover, right IPS is found to work as a bridge between visual cortex and PMA (Alahmadi et al., 2015), furtherly validating the notion that the manipulation of mental images is governed by control processes located in fronto-parietal networks according to current theories on MI (Corbetta and Shulman, 2002; Ganis and Schendan, 2011).

Taken together, the results related to MI with the dominant hand suggest that unlike HC, CIS patients, to produce accurate images, needed not only more attention, memory and the aid of brain areas deputed to motor control, as well as for RR-MS patients, but also the engagement of cortical regions devoted to somatosensory elaboration to abstract features of movement control such as ideation, representation and timing. It is like that, at the first stages of the disease, patients were still able to adopt all these resources in order to counteract the changes due to the isolated MS event. Conversely, patients with definite MS encountered more difficulties in engaging visual, sensory and high level cortical regions and, consequently, in preserving aspects, such as timing, relevant to the correct imagination of movements. Additional activations of motor cerebral areas could represent an ineffective attempt to compensate this deficit.

The loss of ability to compensate the effect of MS severity on MI is more evident when the results related to the task with the non-dominant hand are considered. RR-MS patients did not activate extra cerebral regions with respect to the other two groups; only CIS patients showed more recruitment of parietal and frontal areas when compared with HC subjects, especially sensory areas (S1 and right SPL) and motor areas deputed to movement preparation, selection (PMd bilaterally) and execution (M1), defining a pattern of activation similar to that shown by RR-MS patients during MI with dominant hand. Moreover, CIS with respect to RR-MS patients showed more activation of putamen, interconnected with many other structures to control many motor skills such as, for examples, motor learning, motor preparation and movement sequences. Results suggest that more challenging is the task, more difficult the recruitment of compensatory brain areas, with a consequent deficit in behavioral performance. Thus, the neuroimaging results may explain the differences found in anisochrony among groups and between dominant and non-dominant hand.

These considerations are further supported by results from voxelwise correlation analysis and comparison between MM and AM. The former showed that, during MI with right hand, increased activity in sensory areas correlated with the increment of IP in the whole patient group. Moreover, worse performance according to the IP correlated with activations in cerebellum and several frontal areas usually involved in motor control in RR-MS group. In addition, voxelwise correlation analysis confirmed the shift of activation depending on the disease stage and complexity of the task. Indeed, for non-dominant hand increased cerebellar activations was again associated with higher IP in RR-MS, whereas a relationship between IP and controlateral frontal regions (right dorsolateral prefrontal cortex) was observed in CIS group when considered alone; negative correlations with other regions involved in motor control were found in ipsilateral frontal areas (left medial prefrontal cortex and left IFG) and basal ganglia (left putamen and pallidum). The associations between IP and MI activation in the patient groups are more distributed and distinct from those previously observed in healthy participants, where worse performance was associated with higher activation in the left superior parietal lobe and better performance was related to increased right prefrontal cortex recruitment (Saiote et al., 2016).

Similarly, the comparison of MM and AM showed that CIS patients activated a more extensive MI network than HC for both dominant and non-dominant side, involving frontal, parietal and subcortical regions. Moreover, they showed a higher activation difference between MM and AM with respect to RR-MS group in regions sub-serving sensory processing and higher level functions. RR-MS differed from HC group in MI with dominant hand principally activating brain structures mainly involved in motor control, whereas, as expected from above considerations, no differences were found between these two groups for the non-dominant hand.

In conclusion, we found that MS patients with different disease courses showed different pattern of neural engagement during MI and that the networks of brain activations depended on the hand used to execute/imagine the movement. The association between brain activations and anisochrony is reflected in the IP suggesting that it could be considered as a personalized measure of actual and mental execution performance, correlated with disease severity. Therefore, due to the MS impact on motor and cognitive domains already after the first attacks, an indicator such as the IP, taking into account not only motor performance but also cognitive and mental aspects, could be useful as a surrogate behavioral marker of MS severity, especially in the early stages of the disease.

## Author Contributions

AT and CS: substantial contribution to the conception, design, acquisition, analysis and interpretation of the work; drafted and then revised the work after the revision of the coauthors; finally, approved the version to be published; agree to be accountable for all aspects of the work in ensuring that questions related to the accuracy or integrity of any part of the work are appropriately investigated and resolved. GBr, CC, MAB and GLM: substantial contribution to the interpretation of the work; revised the work; finally, approved the version to be published; agree to be accountable for all aspects of the work in ensuring that questions related to the accuracy or integrity of any part of the work are appropriately investigated and resolved. GBo and LR: substantial contribution to the acquisition and analysis of the work; revised the work; finally, approved the version to be published; agree to be accountable for all aspects of the work in ensuring that questions related to the accuracy or integrity of any part of the work are appropriately investigated and resolved. MI: substantial contribution to the conception, design, acquisition, analysis and interpretation of the work; revised the work; finally, approved the version to be published; agree to be accountable for all aspects of the work in ensuring that questions related to the accuracy or integrity of any part of the work are appropriately investigated and resolved.

## Conflict of Interest Statement

The authors declare that the research was conducted in the absence of any commercial or financial relationships that could be construed as a potential conflict of interest.

## Footnotes

^1^http://afni.nimh.nih.gov

## Abbreviations

9HPT: Nine-Hole Peg Test
AM: actual squeezing ball movement
CIS: clinically isolated syndrome
CST: cortico-spinal tract
EDSS: Edinburgh Handedness Inventory Scale
HC: healthy controls
IFG: inferior frontal gyrus
IP: Index of performance
IPS: intra-parietal sulcus
KVIQ: Kinesthetic and Visual Imagery Questionnaire
LOC: lateral occipital cortex
M1: primary motor area
MFG: middle frontal gyrus
MFIS: Modified Fatigue Impact Scale
MM: mental squeezing ball movement
MS: multiple sclerosis
PMA: premotor area
PMd: dorsal premotor
PPL: posterior parietal lobe
RR-MS: relapsing-remitting multiple sclerosis
S1: primary sensory area
SDMT: Symbol Digit Modalities Test
SFG: superior frontal gyrus
SMA: supplementary motor area
SPL: superior parietal lobule
T2LV: T2 hyperintense lesion volume.
