# Supplementary material for: Motor Imagery as a Function of Disease Severity in Multiple Sclerosis: An fMRI Study
Source: Front Hum Neurosci. 2018 Jan 11;11:628. doi: 10.3389/fnhum.2017.00628 (PMC5768615; doi:10.3389/fnhum.2017.00628)
Supplement: Supplementary file 1 [file Table_1.docx]

Supplementary Material

Motor imagery as a function of disease severity in multiple sclerosis: An fMRI study.

Andrea Tacchino, Catarina Saiote, Giampaolo Brichetto, Giulia Bommarito, Luca Roccatagliata, Christian Cordano, Mario Alberto Battaglia, Gian Luigi Mancardi and Matilde Inglese

*** Correspondence:** Matilde Inglese, matilde.inglese@mssm.edu

# Supplementary Table 1

|  | **AM_R**  **(Mean ± SD)** | **MM_R**  **(Mean ± SD)** | **AM_L**  **(Mean ± SD)** | **MM_L**  **(Mean ± SD)** |
| --- | --- | --- | --- | --- |
| **RR-MS** | 26.10 ± 9.33 | 18.26 ± 8.06 | 27.03 ± 8.51 | 17.83 ± 6.65 |
| **CIS** | 28.32 ± 9.28 | 22.12 ± 7.90 | 29.47 ± 10.52 | 22.09 ± 8.24 |
| **HC** | 27.51 ± 12.72 | 22.99 ± 9.67 | 27.78 ± 13.05 | 23.04 ± 9.08 |

**Table S1.** Data represent the number of actual squeezing ball movements and the mental squeezing ball movements for right and left hands respectively (AM_R, MM_R; AM_L, MM_L)
